# Income-based disparities in the risk of distant-stage cervical cancer and 5-year mortality after the introduction of a National Cancer Screening Program in Korea

**DOI:** 10.4178/epih.e2022066

**Published:** 2022-08-11

**Authors:** Erdenetuya Bolormaa, Seung-Ah Choe, Mia Son, Myung Ki, Domyung Paek

**Affiliations:** 1 Department of Public Health, Korea University, Seoul, Korea; 2 Division of Life Sciences, Korea University, Seoul, Korea; 3 Department of Preventive Medicine, Kangwon National University School of Medicine, Chuncheon, Korea; 4 Department of Preventive Medicine, Korea University College of Medicine, Seoul, Korea; 5 Wonjin Institute for Occupational and Environmental Health, Green Hospital, Seoul, Korea; 6 Graduate School of Public Health, Seoul National University, Seoul, Korea

**Keywords:** Income, Socioeconomic factors, Cervical cancer, Survival, Mortality

## Abstract

**OBJECTIVES:**

This study assessed the socioeconomic gradient in the risk of distant-stage cervical cancer (CC) at presentation and 5-year mortality for new CC patients after the introduction of a national Cancer Screening Program (NCSP) in Korea.

**METHODS:**

All new CC cases from 2007 to 2017 were retrieved from the Korea Central Cancer Registry database linked with the National Health Information Database of the National Health Insurance Service. The age-standardized cumulative incidence of CC, adjusted odds ratios (ORs) of distant metastasis at presentation, and adjusted all-cause mortality hazard ratios (HRs) within 5 years post-diagnosis were assessed according to the income gradient.

**RESULTS:**

The 11-year age-standardized cumulative incidence of CC ranged from 48.9 to 381.5 per 100,000 women, with the richest quintile having the highest incidence. Of 31,391 new cases, 8.6% had distant metastasis on presentation, which was most frequent among Medical Aid beneficiaries (9.9%). Distant-stage CC was more likely when the income level was lower (OR, 1.46; 95% confidence interval [CI]), 1.28 to 1.67 for the lowest compared to the richest) and among Medical Aid beneficiaries (OR, 1.50; 95% CI, 1.24 to 1.82). The 5-year mortality was greater in the lower-income quintiles and Medical Aid beneficiaries than in the richest quintile.

**CONCLUSIONS:**

The incidence of CC was higher in the richest quintile than in the lower income quintiles, while the risk of distant-stage CC and mortality was higher for women in lower income quintiles in the context of the NCSP. A more focused approach is needed to further alleviate disparities in the timely diagnosis and treatment of CC.

## INTRODUCTION

Cervical cancer (CC) is the fourth most common cancer and one of the leading causes of cancer death in women, with an estimated 604,000 cases and 342,000 deaths reported in 2020 [1]. Numerous young and less-educated women in society’s poorest populations suffer from this disease [2]. The mortality rate of CC has declined in high-income countries in recent decades, owing to readily accessible cancer screening programs and timely treatment [3-5]. Although affordable screening programs have been widely implemented, a lower socioeconomic status (SES) is associated with a 2-times or 3-times greater risk of CC incidence [6-11]. Despite the reported overdiagnosis of early-stage cancer with increased screening coverage [12], women in lower socioeconomic strata generally show higher CC-specific mortality [13]. The higher allcause and CC-specific mortality in people in lower socioeconomic positions have been explained by inequitable disparities in screening practices, leading to advanced-stage cancer at presentation [14,15].

The crude incidence of CC in Korea decreased from 19.7 in 2013 to 18.9 in 2016 per 100,000 women [16], although it is higher than that in other high-income countries of the Asia-Pacific region, including Japan, New Zealand, and Australia [17], and the global target of less than 4 per 100,000 [18]. Since 2002, Korea has been conducting a national cytology screening program as part of the National Cancer Screening Program (NCSP) for women aged 30 years and above whose incomes are in the bottom 50% of the scale [19]. The screening rate for CC has steadily increased from 54.8% in 2005 to 65.6% in 2015 [20].

Research on the process of cancer development encompasses several dimensions, such as screening, incidence, and mortality. These dimensions are conceptually independent, but they are interrelated. Early diagnosis predicts better survival, in particular when a cancer such as CC slowly grows. Likewise, socioeconomic disparities in mortality reflect inequalities in incidence, the stage at diagnosis, and treatment. Socioeconomic inequalities at one stage of the cancer continuum can be translated into inequalities in the next, highlighting the importance of examining multiple dimensions of cancer. However, previous studies regarding the socioeconomic patterns of cancer mostly focused on a single aspect of cancer management—screening [18,19], incidence [7,10], and mortality [21,22]— as if they are separate phenomena. Given the less optimal screening among women of lower SES, women with lower income levels are more likely to present with advancedstage cancer, even in the context of the NCSP [20,23,24]. A higher risk of advanced cancer in the low-income strata would reduce survival odds following diagnosis. To tackle the socioeconomic disparity in CC mortality, identifying points where a public health program can effectively intervene is critical. A universal cancer screening program is a public health approach to reduce this disparity. Although some researchers have suggested that disparities can be reduced by improving the accessibility of cancer screening tests [25], it has not been elucidated whether the socioeconomic disparity in the timely diagnosis of CC is reflected in a disparity in mortality in the setting of the NCSP. We present socioeconomic disparities in the incidence, stage at presentation, and mortality during the period of universal screening implementation in Korea to understand the pattern of income-based inequalities among CC patients in the cancer continuum.

## MATERIALS AND METHODS

### Data

Based on the International Classification of Diseases 10th revision (ICD-10) code starting with “C53,” 43,139 new patients were identified as having been diagnosed with CC in the Korea Central Cancer Registry (KCCR) databases from 2004 to 2017. Data on the pseudonymized patient identification number, cancer stage, and date of initial diagnosis were extracted from the database. The KCCR is a population-based cancer registry established in 2000 by the National Cancer Center, covering over 90% of new cancer cases diagnosed inside or outside the organized screening program in Korea. Since 2002, national cancer incidence statistics have been created by merging 11 regional population-based cancer registry programs, *ad hoc* medical record review data, and Statistics Korea’s cancer mortality database [26]. The individual data were then linked to the National Health Information Database of the National Health Insurance Service (NHIS) in Korea, which covers about 96% of the Korean population [27], to construct a national cohort of newly diagnosed CC patients. This national insurance database contains general demographics, including employment status, household income as a percentile, residential district, and clinical information on diagnoses, prescribed medications, procedures, and treatments covered by the national health insurance for each visit to a healthcare facility. Given that we had access to the NHIS database only from 2007 to 2017, we limited our study population to cases from the linked KCCR-NHIS database from 2007 to 2017. The final national cohort of CC patients was 31,397 women.

### Cancer stage at presentation and all-cause mortality

The KCCR database provides summary cancer staging information based on the staging system from the Surveillance, Epidemiology, and End Results (SEER) database of the United States National Cancer Institute. It classifies cancers into localized, regional, and distant stages. The “localized” stage represents no signs of spread outside the cervix, and the “regional” stage extends outside the cervix to surrounding structures. In the “distant” stage, cancer spreads to distant parts of the body, such as the lungs, liver, or bones. An unknown stage was recorded separately as ”unknown” by the physician. The study population was classified into 3 categories (locoregional, distant, and unknown) to assess the risk of advanced cancer at presentation. In the NHIS database, the date of death or the date of the last visit to the healthcare facility was used to estimate the duration of survival or the censored survival period, respectively.

### Relative levels of household income and covariates

We used National Health Insurance (NHI) premium information to indicate SES [26]. The NHI premium can be a useful proxy variable for SES because it is calculated based on the estimated income from employees’ wages (for those covered by “employee insurance”) or assets (“local subscribers”). The Korean NHI cohort database includes individually linked personal income decile data. For people living below the national poverty line, the Medical Aid system, a public assistance program, provides healthcare benefits to low-income families, representing approximately 3% of all nationals. The study population was divided into 6 groups according to their relative income level (first quintile, 80-100%, highest income; second quintile, 70-89%; third quintile, 40-59%; fourth quintile, 20-39%; and fifth quintile, 0-19%, lowest income) and if they were receiving Medical Aid, for analytical convenience.

Explanatory factors for the risk of distant-stage CC at presentation and all-cause mortality were selected based on prior knowledge. We retrieved information on age, income rank, disability registration, employment, and the patient’s residential area from the qualification data of the NHIS database, which is annually updated. The type of cancer, year of diagnosis, and comorbid diseases were coded based on the treatment data. Information on body mass index (BMI) and smoking was available from the health check-up database.

### Statistical analysis

First, descriptive statistics were computed at various presentation stages, with all-cause mortality calculated within 5 years of diagnosis. Second, a test was conducted for a linear tendency across income levels in the risk of CC with distant metastasis at presentation. The total number of women by age group and relative income level within the national population was extracted from the NHI statistical yearbooks [28] to calculate the incidence at the population level. The cumulative incidence was calculated by dividing the total number of incidents for each combination of age and income groups by the corresponding total number of the population. Third, odds ratios (ORs) were calculated to assess the association between income level and the risk of distant-stage CC at presentation, adjusted for women’s age, level of income, disability registration, employment, residence in the Seoul metropolitan area, BMI, smoking, and year of diagnosis using a multivariable logistic regression model. Women’s age was categorized into 6 groups (< 30, 30s, 40s, 50s, 60s, and ≥ 70 years), and BMI was categorized into 4 groups (> 18.5, 18.5-25.0, 25.0-30.0, and ≥ 30.0 kg/m^2^). Information on disability, employment, and current smoking was dichotomized. The year of diagnosis was included as a continuous variable (years). Each patient’s residential area was coded according to whether or not they lived in the Seoul metropolitan area, which is home to half of Korea’s total population. To assess temporal changes in income-based disparities, we compared the risk of distant-stage CC in 2007-2009 with the risks in 2010-2013 and in 2014-2017, adjusting for the linear effect of time (year). Lastly, because there was no cause of death information in the KCCR-NHIS database, adjusted hazard ratios (HRs) of all-cause mortality were calculated within 5 years of diagnosis. We included the effect of time-updated income in the survival analysis using annual income level data. The HRs were adjusted for women’s age, BMI, disability registration, smoking, employment, residential area, year of diagnosis, charlson comorbidity index (CCI), and stage at presentation using a Cox proportional-hazard model. We calculated the CCI based on the ICD-10 coding algorithm of Sundararajan et al. [29]. Given that over 10% of the patients lacked information on BMI (48.0% missing rate) and smoking (32.7%), missing variables were imputed using the fully conditional method, which is a statistically valid method for creating imputations in a large dataset [30]. All analyses were performed using the SAS version 9.4 (SAS Institute Inc., Cary, NC, USA).

### Ethics statement

The analyses are based on a de-identified dataset and thus did not require approval from the Institutional Review Board of Kangwon National University.

## RESULTS

**Figure 1.**
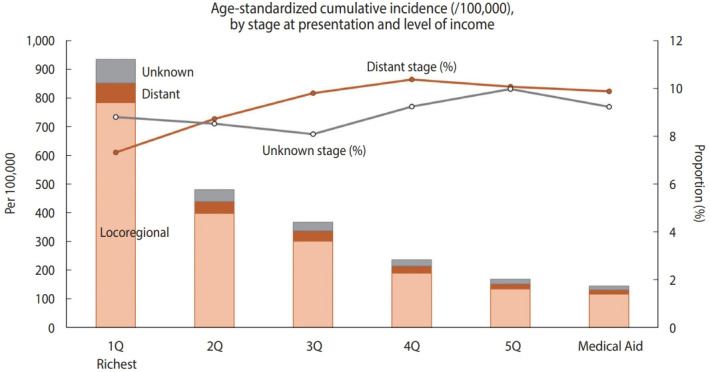
Age-standardized cumulative incidence of cervical cancer for 11 years per 100,000 women by income quintile, Korean Central Cancer Registry linked with the National Health Insurance database in 2007-2017. 1Q, first quintile (richest); 2Q, second quintile; 3Q, third quintile; 4Q, fourth quintile; 5Q, fifth quintile.

During the study period, the annual incidence rate of new CC was 31.5 per 100,000 woman-years on average. Of the new CC patients, 8.6% (n= 2,688) had distant metastasis at the time of diagnosis, and 8.2% (n = 2,583) were registered as having an unknown stage. When stratified by relative income level, the agestandardized cumulative incidence of CC per 100,000 women over the 11-year period (2007-2017) was higher when the relative income was higher, peaking in the richest quintile (933.4 per 100,000 women; Figure 1). Compared to the highest income quintile (7.3%), the lower-income quintiles and Medical Aid beneficiaries showed a higher proportion of distant-stage CC at presentation (10.4% in the fourth quintile, 10.1% in the fifth quintile, and 9.9% in Medical Aid beneficiaries) than those in the richest income quintile.

Of the 31,391 women CC patients, the majority were under the age of 60 (67.5%), non-employed (88.9%), covered by employee health insurance (66.9%), and from relatively higher income groups (27.1% were in the highest quintile and 38.0% were in the third or lower quintile of the national population; Table 1). The majority (83.2%) of patients presented with a local or regionalized SEER stage, while 8.6% had distant metastasis (n= 2,688). An unknown stage was present in 8.2% (n= 2,583). The proportion of patients with distant-stage CC was highest among women aged 70 and over and lowest among women under 30. Compared to health insurance subscribers, Medical Aid beneficiaries showed nearly twice the prevalence of distant and unknown stages. Among the health-insured employees and local subscribers, the distribution of stage at presentation was generally similar in terms of the relative income level. Differences in the distribution of SEER stages according to disability registration, BMI categories, or smoking history were not observed.

**Figure 2.**
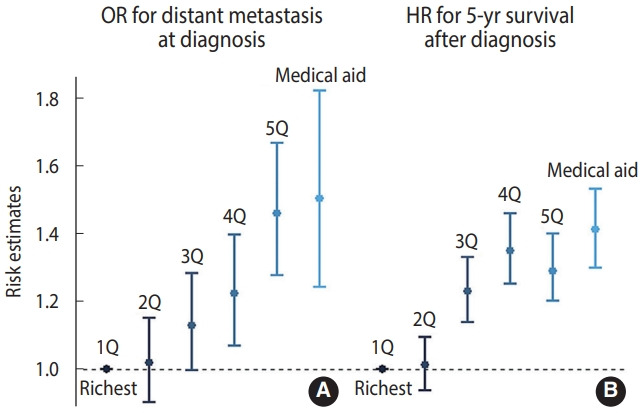
Odds ratios (OR) for distant-stage cancer (A) and hazard ratios (HRs) for all-cause mortality (B) by income level, Korean Central Cancer Registry linked in 2007-2017 (n=28,808 for ORs, n=31,391 for HRs). The OR estimates were adjusted for women’s age, level of income, disability registration, employment, residence in the Seoul metropolitan area, body mass index, smoking, and year of diagnosis. The HRs were further adjusted for comorbidity index and stage (including unknown stage). 1Q, first quintile (richest); 2Q, second quintile; 3Q, third quintile; 4Q, fourth quintile; 5Q, fifth quintile.

For CC patients with available information on stage at presentation, the adjusted OR for the distant stage was higher when the income level was lower (OR, 1.37; 95% confidence interval [CI], 1.13 to 1.67 for Medical Aid beneficiaries; OR, 1.43; 95% CI, 1.25 to 1.64 for the fifth quintile; OR, 1.18; 95% CI, 1.03 to 1.36 for the fourth quintile; and OR, 1.12; 95% CI, 0.99 to 1.27 for the third quintile, compared to the richest quintile), while the OR for the second-highest income quintile versus the richest group was close to 1 (1.02; 95% CI, 0.90 to 1.15; Figure 2). Similarly, the all-cause mortality risk within 5 years was higher at lower incomes (HR, 1.41, 95% CI, 1.30 to 1.53 for Medical Aid beneficiaries; HR, 1.30; 95% CI, 1.20 to 1.40 for the fifth quintile; HR, 1.35; 95% CI, 1.25 to 1.46 for the fourth quintile; and HR, 1.23; 95% CI, 1.14 to 1.33 for the third quintile, compared to the richest quintile). Regarding unknown-stage CC at presentation, the fifth (lowest) income quintile and Medical Aid beneficiaries showed higher risk than the richest quintile (OR, 1.36; 95% CI, 1.13 to 1.63) (Supplementary Material 1). The extent of income-based disparities in the risk of distant-stage CC did not show a significant change (2007-2009 vs. 2010-2013, p = 0.668; 2007-2009 vs. 2014-2017, p = 0.404) (Supplementary Material 2).

## DISCUSSION

Using data from the National Cancer Registry for 11 years, we observed a higher incidence of CC and a lower risk of distant stage at presentation when income levels were higher. Lower-income levels and being a Medical Aid beneficiary were consistently associated with a higher risk of distant-stage CC at presentation and a higher risk of all-cause mortality within 5 years of diagnosis after adjusting for individual risk factors. This pattern of association was also observed for the risk of unknown-stage CC. This finding supports our hypothesis of a higher risk of advanced CC at presentation in persons with a lower SES, which inevitably results in higher mortality following diagnosis. This study adds empirical evidence for a positive association between the cancer stage at presentation and the relative income level in CC patients when an NCSP is implemented.

Based on prior knowledge of risk factors for CC [11,31,32], the higher incidence of CC among richer women is counterintuitive. This finding may be attributed to differential compliance with the CC screening program. Even with the free NCSP, women with a lower SES would have limited access to resources required for disease prevention (such as human papillomavirus [HPV] immunization) or health promotion activities (preventing cervical infection, quitting smoking, etc.) [23,33,34]. In addition to the higher baseline risks, a lack of knowledge about the effectiveness of regular cancer screening in low-income quintiles might have led to reduced access to care, which is also affected by financial, physical, and social barriers. A lack of timely information about recommended guidelines for cancer screening, lack of financial resources to enable routine screening, and employment status were major barriers to screening among women with low SES [23]. The higher probability of advanced CC at presentation among people with low incomes indicates that relatively low income is associated with less optimal screening performance. The Unite States data for 2 decades showed that patients in census tracts with lower SES had significantly higher rates of late-stage CC diagnosis and lower survival than those living in areas with higher socioeconomic conditions [8]. Women with private health insurance had earlier stage disease at presentation than those with federal health insurance or no insurance at all [15]. In a national study of Danish CC patients, shorter duration of education, lower incomes, and living without a partner were associated with lower survival, which was explained mainly by the cancer stage at presentation [13].

The association between lower SES and higher all-cause mortality has been consistently observed in numerous studies, regardless of whether an NCSP is implemented [8,13,22,35]. Among Danish CC patients, all-cause mortality was highest in the lowest-income group after adjusting for comorbidity and cancer stage at presentation [13]. In the United States, women living in lower-SES regions had significantly lower survival rates when diagnosed, even when their CC stage at presentation was early [16]. In a study of Korean women, CC mortality was higher among less-educated and unmarried women [36]. In a spatial analysis of Germany, with a drop in social status, clusters showed a 60% increase in mortality relative to the highest social class. Lifestyle, participation in screening programs, exposure to carcinogens, and environmental exposure would be some of the explanations for the relationship between social status and cancer mortality [37]. Another explanation concerns a pathway through which socioeconomic inequalities in the CC stage at presentation can be transmitted to inequalities in all-cause mortality.

The findings of this study must be interpreted with caution. Since CC has a long latency period, ranging from 10 years to 20 years, when assessing the relationship between SES and incidence, the income level at the time of CC diagnosis may not reflect the socioeconomic position at the beginning of cancer development. Given the potential change in the covariates over time, in addition, the failure to consider time-updated values of BMI or smoking in the model may limit the interpretation of our findings. Because the health check-ups covered by the NHI are conducted every 2 years for generally healthy people, many of the patients did not have BMI or smoking information during the post-diagnosis period. We believe that the inclusion of the time-updated income level in our survival model would reflect some change in women’s general health conditions. Second, residual confounding effects from unmeasured variables such as diet, education, occupational exposure, and HPV immunization status would have biased our findings. The effect of HPV immunization on CC incidence during the study period would have been minimal because the vaccine was first introduced in 2006 and implemented in Korea’s national immunization program in 2016. Using data from the National Cancer Registry, we believe our findings can provide insights into socioeconomic disparities in the timely diagnosis and survival of CC, in a context where an NCSP is implemented for half of the population.

Lower-income levels and medical beneficiaries were associated with a higher risk of distant-stage CC at presentation and higher all-cause mortality, despite implementing an NCSP for low-income populations. A more focused approach beyond the free screening program is needed to address the income-based disparity in the diagnosis and survival of CC patients.

## Figures and Tables

**Table 1.** Demographic characteristics and SEER stage at presentation in new CC patients, Korean Central Cancer Registry linked with the National Health Insurance database in 2007-2017 (n=31,391)

Characteristics	Locoregional (n=26,120)	Distant (n=2,688)	Unknown (n=2,583)	Total (n=31,391)	p for heterogeneity
Age (yr)					
<30	727 (86.7)	28 (3.3)	84 (10.0)	839	<0.001
30-39	4,235 (88.7)	221 (4.6)	321 (6.7)	4,777	
40-49	7,243 (87.3)	568 (6.9)	484 (5.8)	8,295	
50-59	6,122 (84.2)	676 (9.3)	471 (6.5)	7,269	
60-69	3,916 (82.5)	456 (9.6)	374 (7.9)	4,746	
≥70	3,877 (70.9)	739 (13.5)	849 (15.5)	5,465	
Employment status					
Employed	3,117 (89.2)	176 (5.0)	201 (5.8)	3,494	<0.001
Non-employed	23,003 (82.5)	2,512 (9.0)	2,382 (8.5)	27897	
Relative income (quintile)^1^					
1Q (highest)	7,125 (83.7)	699 (8.2)	687 (8.1)	8,511	<0.001
2Q	5,406 (84.8)	490 (7.7)	479 (7.5)	6,375	
3Q	4,714 (85.0)	453 (8.2)	383 (6.9)	5,550	
4Q	3,990 (84.1)	389 (8.2)	366 (7.7)	4,745	
5Q (lowest)	3,185 (80.3)	396 (10.0)	384 (9.7)	3,965	
Medical Aid	892 (70.8)	169 (13.4)	199 (15.8)	1,260	
Disability registration					
Any disability	1,635 (75.3)	237 (11.0)	299 (13.8)	2,171	<0.001
No disability	24,410 (83.8)	2,440 (8.4)	2,269 (7.8)	29,119	
Region					
Seoul metropolitan area^2^	12,761 (84.2)	1,255 (8.3)	1,134 (7.5)	15,150	<0.001
Outside of Seoul metropolitan area	13,359 (82.3)	1,433 (8.8)	1,449 (8.9)	16,241	
Body mass index (kg/m^2^)					
<18.5	833 (84.8)	61 (6.2)	88 (9.0)	982	<0.001
18.5-25.0	17,300 (83.7)	1,726 (8.3)	1,643 (8.0)	20,669	
25.0-30.0	7,401 (81.9)	831 (9.2)	800 (8.9)	9,032	
≥30.0	586 (82.8)	70 (9.9)	52 (7.4)	708	
Smoking					
Non-smoker	24,232 (83.0)	2,493 (8.6)	2,437 (8.4)	29,162	0.011
Past or current smoker	1,888 (84.7)	195 (8.7)	146 (6.6)	2,229	

Values are presented as number (%). SEER, Surveillance, Epide miology, and End Results; CC, cervical cancer.

1 1Q, first quintile; 2Q, second quintile; 3Q, third quintile; 4Q, fourth quintile; 5Q, fifth quintile.

2 Seoul, Incheon, and Gyeonggi Province, located in northwestern Korea.

## SUPPLEMENTARY MATERIALS

Supplementary Material 1. Adjusted odds ratios for unknown stage at presentation across relative level of income, National Cancer Registry of Korea linked with the National Health Information Database of National Health Insurance database in 2007-2017 (n=31,391). Estimates are adjusted for age, body mass index, employment, disability, year of diagnosis and Seoul metropolitan area residence. OR, odds ratio; 1Q, 1^st^ quintile (richest); 2Q, 2^nd^ quintile; 3Q, 3^rd^ quintile; 4Q, 4^th^ quintile; 5Q, 5^th^ quintile.

Supplementary Material 2 Adjusted odds ratios for advanced stage at presentation across relative level of income by year of cervical cancer diagnosis, National Cancer Registry of Korea linked with the National Health Information Database of National Health Insurance database in 2007-2017 (n=31,391). OR, odds ratio; 1Q, 1^st^ quintile (richest); 2Q, 2^nd^ quintile; 3Q, 3^rd^ quintile; 4Q, 4^th^ quintile; 5Q, 5^th^ quintile. Estimates are adjusted for age, body mass index, employment, disability, and Seoul metropolitan area residence.
