# Supplementary material for: Income-based disparities in the risk of distant-stage cervical cancer and 5-year mortality after the introduction of a National Cancer Screening Program in Korea
Source: Epidemiol Health. 2022 Aug 11;44:e2022066. doi: 10.4178/epih.e2022066 (PMC10089710; doi:10.4178/epih.e2022066)
Supplement: Supplementary Material 1. — Adjusted odds ratios for unknown stage at presentation across relative level of income, National Cancer Registry of Korea linked with the National Health Information Database of National Health Insurance database in 2007-2017 (n=31,391). Estimates are adjusted for age, body mass index, employment, disability, year of diagnosis and Seoul metropolitan area residence. OR, odds ratio; 1Q, 1st quintile (richest); 2Q, 2nd quintile; 3Q, 3rd quintile; 4Q, 4th quintile; 5Q, 5th quintile. [file epih-44-e2022066-suppl1.docx]

**
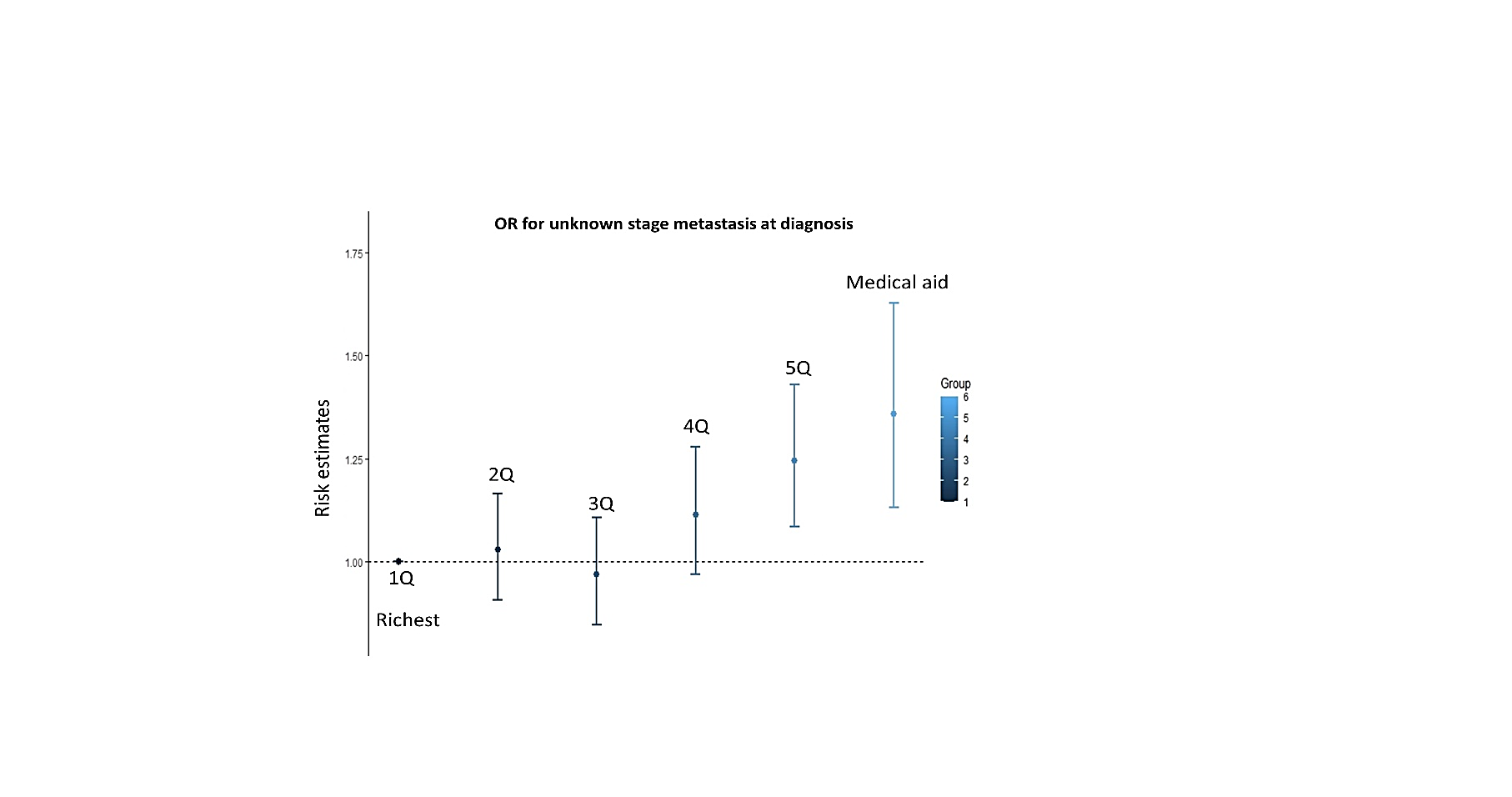
**

**Supplementary Material 1.** Adjusted odds ratios for unknown stage at presentation across relative level of income, National Cancer Registry of Korea linked with the National Health Information Database of National Health Insurance database in 2007-2017 (n=31,391). Estimates are adjusted for age, body mass index, employment, disability, year of diagnosis and Seoul metropolitan area residence. OR, odds ratio; 1Q, 1^st^ quintile (richest); 2Q, 2^nd^ quintile; 3Q, 3^rd^ quintile; 4Q, 4^th^ quintile; 5Q, 5^th^ quintile.
