# Supplementary material for: Income-based disparities in the risk of distant-stage cervical cancer and 5-year mortality after the introduction of a National Cancer Screening Program in Korea
Source: Epidemiol Health. 2022 Aug 11;44:e2022066. doi: 10.4178/epih.e2022066 (PMC10089710; doi:10.4178/epih.e2022066)
Supplement: Supplementary Material 2 — Adjusted odds ratios for advanced stage at presentation across relative level of income by year of cervical cancer diagnosis, National Cancer Registry of Korea linked with the National Health Information Database of National Health Insurance database in 2007-2017 (n=31,391). OR, odds ratio; 1Q, 1st quintile (richest); 2Q, 2nd quintile; 3Q, 3rd quintile; 4Q, 4th quintile; 5Q, 5th quintile. Estimates are adjusted for age, body mass index, employment, disability, and Seoul metropolitan area residence. [file epih-44-e2022066-suppl2.docx]

**
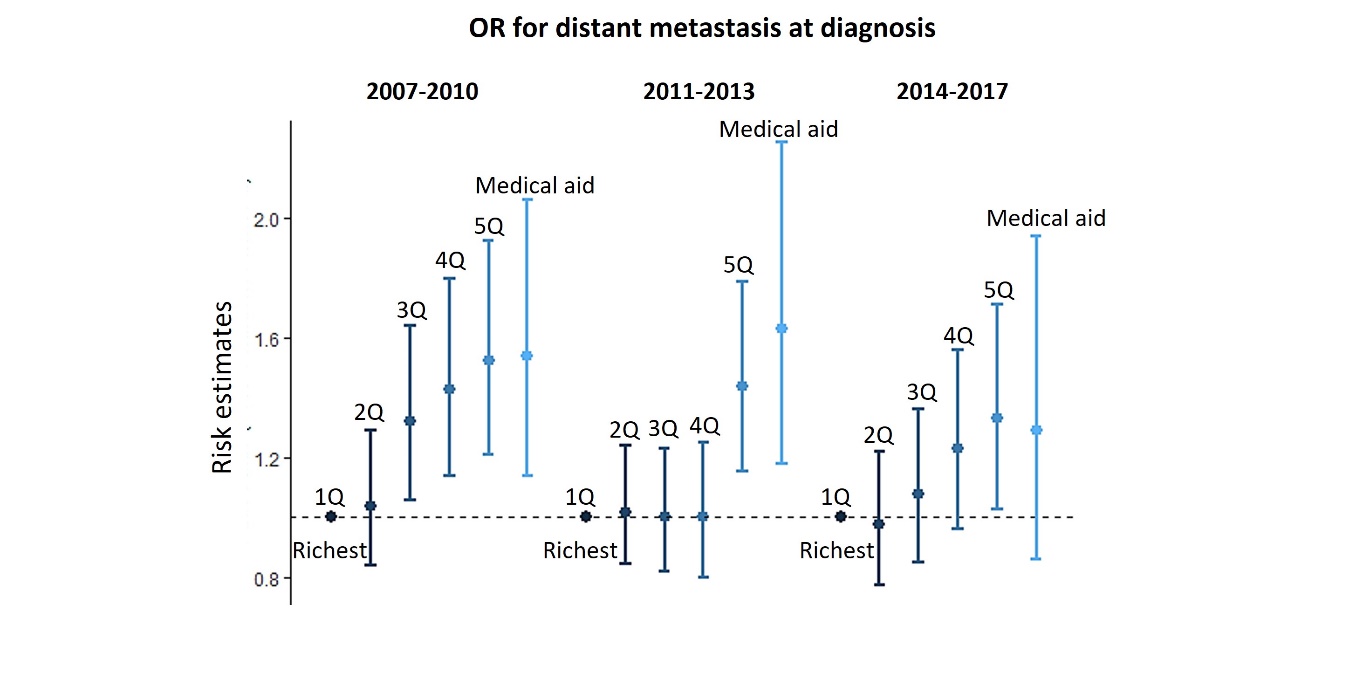
**

**Supplementary Material 2.** Adjusted odds ratios for advanced stage at presentation across relative level of income by year of cervical cancer diagnosis, National Cancer Registry of Korea linked with the National Health Information Database of National Health Insurance database in 2007-2017 (n=31,391). OR, odds ratio; 1Q, 1^st^ quintile (richest); 2Q, 2^nd^ quintile; 3Q, 3^rd^ quintile; 4Q, 4^th^ quintile; 5Q, 5^th^ quintile. Estimates are adjusted for age, body mass index, employment, disability, and Seoul metropolitan area residence.
